# Tracking Visual Events in Time in the Absence of Time Perception: Implicit Processing at the ms Level

**DOI:** 10.1371/journal.pone.0127106

**Published:** 2015-06-01

**Authors:** Patrick Eric Poncelet, Anne Giersch

**Affiliations:** INSERM U1114, Department of Psychiatry, Fédération de Médecine Translationnelle de Strasbourg, Strasbourg University Hospital, Strasbourg, France; University of Muenster, GERMANY

## Abstract

Previous studies have suggested that even if subjects deem two visual stimuli less than 20 ms apart to be simultaneous, implicitly they are nonetheless distinguished in time. It is unclear, however, how information is encoded within this short timescale. We used a priming paradigm to demonstrate how successive visual stimuli are processed over time intervals of less than 20 ms. The primers were two empty square frames displayed either simultaneously or with a 17ms asynchrony. The primers were followed by the target information after a delay of 25 ms to 100 ms. The two square frames were filled in one after another with a delay of 100 ms between them, and subjects had to decide on the location of the first of the frames to be filled in. In a second version of the paradigm, only one square frame was filled in, and subjects had to decide where it was positioned. The influence of the primers is revealed through faster response times depending on the location of the first and second primers. Experiment 1 replicates earlier results, with a bias towards the side of the second primer, but only when there is a delay of 75 to 100 ms between primers and targets. The following experiments suggest this effect to be relatively independent of the task context, except for a slight effect on the time course of the biases. For the temporal order judgment task, identical results were observed when subjects have to answer to the side of the second rather than the first target, showing the effect to be independent of the hand response, and suggesting it might be related to a displacement of attention. All in all the results suggest the flow of events is followed more efficiently than suggested by explicit asynchrony judgment studies. We discuss the possible impact of these results on our understanding of the sense of time continuity.

## Introduction

The present time and the sense of time continuity remain difficult concepts to grasp, resulting in a well-known paradox. We experience time as a continuous flow, producing a sense of time continuity, and yet we also experience a sense of present time, usually defined as a temporal window during which all events are deemed to be simultaneous [1,2]. Since all events are judged to be occurring at the same time, it is as if, subjectively, time is at a standstill during this time period. This is incompatible with the concept of time continuity and results in a time paradox. However, the concepts of present time and temporal windows derive from experiments based on subjective judgments and thus overlook automatic coding, i.e. the unconscious coding of information over time. By taking such coding into account, our recent results challenge the idea that all events are processed simultaneously within temporal windows and suggest, on the contrary, that short asynchronies are processed, unbeknown to the subjects concerned [3–5](Giersch, Assche, & Elliott, 2013; Lalanne, van Assche, & Giersch, 2012a; Lalanne, et al., 2012b). We reached this conclusion by measuring implicit effects which, by definition, were independent of a subjective judgment. However, these implicit effects were observed more than 500 ms after the display of target information. We still need to understand how events’ time structure is coded on an implicit level within the first hundred ms following the events’ occurrence, and to which extent this processing involves automatic or attentional mechanisms. Here, we devised a new paradigm aimed at unraveling the time course of the processing of events displayed in close succession and interpreted as being simultaneous. We then discuss the implications of our results for understanding the sense of time continuity.

### The temporal window concept—the subjective present

The temporal window concept has a long history dating back to the 19th century with the concept of the specious present [6]. This history is reviewed elsewhere [1,2,7], and so we shall only present a brief summary of certain aspects relevant to our study. Phenomenologists like Husserl proposed that at the present time, past, present and future information are all present in our mind at once, giving the subjective present [8]. Importantly, this subjective present is thought to have a duration (of around 2–3 s), hence the concept of time windows, in this case ‘subjective windows’. It is a concept which has since been applied to more elementary moments, i.e. ‘functional windows’, within which, according to the typical definition, all information is processed as co-temporal. Since there is a limit to our ability to distinguish events in time, functional windows are thought to have a duration, like subjective windows, only smaller, of around 50 ms. It has been proposed that functional windows are embedded within larger windows, and that this hierarchy [2,9] and overlap of time windows is what leads to the sense of time continuity. However, as already pointed out, the concept of elementary time windows is based on experiments in which subjects are explicitly told to decide whether or not two stimuli are simultaneous, i.e. they have to make an explicit temporal judgment. Our own previous results suggest that events are better distinguished in time than previously thought, on an implicit level [4,5]. Moreover, they also suggest that events are not only distinguished, but that the last event in a sequence of two is privileged after the occurrence of the events. This would mean that events are processed serially even when being judged as being simultaneous. In other words, it would mean that the processing of information is more continuous than suggested by studies measuring elementary time windows. However, the time course of the events processing remains to be explored. It remains also to be checked whether the mechanisms underlying this effect are related to a visuomotor reflex or an attention displacement, and whether it is the second event that is privileged, or the first event that is ignored.

### The temporal window concept—previous evidence of implicit asynchrony coding within the temporal window

On an experimental level, it has repeatedly been shown that there is a limit to subjects’ ability to detect an asynchrony or a temporal order between two successive stimuli [1,2,7,10–12]. Our own results replicated these results, showing that subjects were unable to distinguish between simultaneous and asynchronous visual stimuli when the asynchrony was below 20 ms. Indeed, the rate of ‘simultaneous’ responses was the same for 17 ms asynchronies and for perfect synchrony [5]. Despite this, we obtained indirect evidence that these signals are distinguished in time. We did so by using the Simon effect, defined as a trend to press manually to the side of the displayed stimulus [13–15]. It produces faster responses and fewer errors when the stimulus and response are on the same side, irrespective of the task at hand. For example, if the task is to discriminate shapes, where the shape is located is irrelevant but will nonetheless bias the subject’s response, in that he or she will tend to hit the response key that is on the same side as the shape, regardless of the correct response. Obtaining a Simon effect with our paradigm was not straightforward, given that a stimulus was displayed on both the right and left side of the screen. Indeed, a Simon effect was impossible when the two stimuli were strictly simultaneous, since information in that case was exactly the same on both sides of the screen. However, we observed a Simon effect in the case of asynchrony. Subjects’ responses were systematically biased to the side of the second stimulus, whether it is right or left side. Interestingly, this was true even for asynchronies of less than 20 ms, i.e. for those not distinguished from perfect simultaneity. These results show that visual stimuli were distinguished in time on an implicit level, i.e. when asynchronies were too short to yield a subjective judgment. Second, they suggest that part of the temporal order was also encoded, insofar as if there had only been a coding of asynchrony, independently of the temporal order, there should have been no distinction possible between the first and second stimulus. Hence it would have been impossible to observe a selective bias towards the second stimulus. On the contrary, the existence of the bias towards the second stimulus indicated that the latter had been distinguished from the first stimulus and identified as the second. Hence, it meant that priority was given towards the last occurring stimulus.

Although the bias towards the second stimulus has been replicated in a number of studies, our method of analysis has several limitations, the main one being that the Simon effect can only give indirect indications about the implicit coding of the temporal structure of events. It relies on a visuomotor effect, which might occur early on after the display of the stimuli [16,17], but is observed late, at the time of the response. Such behavioral recordings do not allow access to the time course of the effect. Second, the mechanisms of the Simon effect are still under discussion (see reviews and discussions on the Simon effect in [13–15]). In our paradigm also, it is unclear whether the mechanisms underlying the bias effect are based on automatic visuomotor or more attentional effects. For all these reasons, we devised here a new paradigm aimed at testing the implicit coding of the temporal structure of events as directly as possible. Two stimuli are used as primers, namely square frames displayed to right and left of the screen with a 17 ms onset asynchrony. Thus, participants cannot consciously perceive the asynchrony between the primers. The frames are subsequently filled in, with the content representing the target information. In one version of our paradigm, the frames are filled in in turn, with a clear asynchrony (100 ms), and subjects have to decide on which side a frame had been filled in first (temporal order judgment task). In another version of our paradigm, only one frame is filled in, and subjects have to decide on which side the frame has been filled (detection task). Control experiments are carried out as to whether the invisible asynchrony of the primers influences subsequent judgments made about the target information.

First, in the detection task, we expected to replicate earlier results with a bias to the side of the second primer stimulus. Since the tasks are very easy, we expected this bias to be reflected in faster response times when the target is located on the side of the second primer rather than on the first primer (i.e. when primers are asynchronous). Control conditions with simultaneous primers were designed to measure baseline performance and check whether the modulation of response times corresponds to a facilitation or an impairment effect.

The results predictions in the case of temporal order judgment were more difficult. In this task, two targets are displayed successively, and the additional target induces a perception of direction, which is either congruent or non congruent with the primers direction. It has been shown that the motion direction can be primed even for short duration exposures [18] or when the motion of the primers is not seen consciously [19]. Our primers could not induce an explicit sense of direction (left-right or right-left) since presented below asynchrony detection threshold. However, they could have induced an implicit sense of direction. In that case performance may have been facilitated when primers and targets appeared in the same order (both right-left or both left-right). There is an alternative possibility, though. The bias towards the second primer could represent a phenomenon akin to an attention shift, with the second primer playing the role of an attention cue. This was expected to lead to a facilitation effect when the first target was on the side of the second primer, i.e. when primers and targets appeared in the opposite order (one right-left and the other left-right). The prior entry effect [20] indeed shows that an attentional cue displayed before target onset can facilitate temporal order judgment. This happens if the cue directs attention towards the first target stimulus in a sequence of two. This might thus account for temporal order judgments about targets being easier when the first target appears to the side of the second primer (when target and primer appear in reverse order). The latter possibility, i.e. a bias to the side of the second primer, would be akin to the phenomenon expected in the detection task. The comparison between the two tasks (temporal order judgment vs. detection task) thus allows us to question whether the effect is similar when the main task involves a temporal judgment or not.

All in all, in Experiment 1 we aimed at replicating our earlier results showing that two stimulus presented with a stimulus onset asynchrony of 17 ms generate a bias to the side of the second stimulus. This replication within the framework of a priming paradigm allowed us first to explore the time course of this effect, by manipulating the onset asynchrony between primers and targets, and to check to which amount effects were similar or different across tasks (temporal order judgment vs. detection task).

## Material and Methods

### General Method

The project was approved by the local ethics committee (People protection Committee "Est-IV”). All subjects gave their informed written consent prior to testing, in accordance with the recommendations laid down in the Helsinki Declaration.

#### Participants

The following experiments were conducted with 60 healthy volunteers (38 women and 22 men). There were 12 participants per Experiment (except in Experiment 4, which involved two different groups of subjects) (see Table 1 for more details). All of them were students at the University of Strasbourg aged between 19 and 28.

**Table 1:** Overview of the mean age of the groups of subjects participating in the four experiments.

	Experiment 1	Experiment 2	Experiment 3	Experiment 4	
	TOJ/Detection 25/50/75/100 ms	Detection 100 ms	Detection 50 ms	TOJ 100	
**Groups’ mean age**	23,4	21,5	24,4	24,1	23,6
**SD**	3,2	2,1	2,2	2,5	2,8

The subjects were all unaware of the specific aims of the experiment and were not given more detailed information until after the experimental session. All the participants had normal or corrected-to-normal visual acuity (greater than 0.8, checked with the Snellen’s scale). None of them had any neurological or serious somatic disease or had taken any narcotics or drugs affecting the CNS.

#### Equipment

The experiments were conducted on a DELL Dimension 4600 computer. The whole experimental protocol was developed using MATLAB software (R2007a) by MathWorks and CRS VSG Toolbox for MATLAB. The visual stimuli were presented on a 20” 120 Hz CRT screen (resolution 800 * 600) with a visual stimulus generator ViSaGe (Visual Stimulus Generator, Cambridge Research Systems) which meant it was possible to control the contrast and exposure delays of the stimuli. Subjects had to put their head on a chin rest placed 1.00 m from the screen. Each pixel on the screen represented an angular size of 0.03° of visual angle, and the stimuli luminance was increased by steps of 8.33 ms. Subjects responded by pressing with their thumbs on one of two response buttons on a box that recorded response times with millisecond accuracy.

#### Stimuli

**Fig 1:**
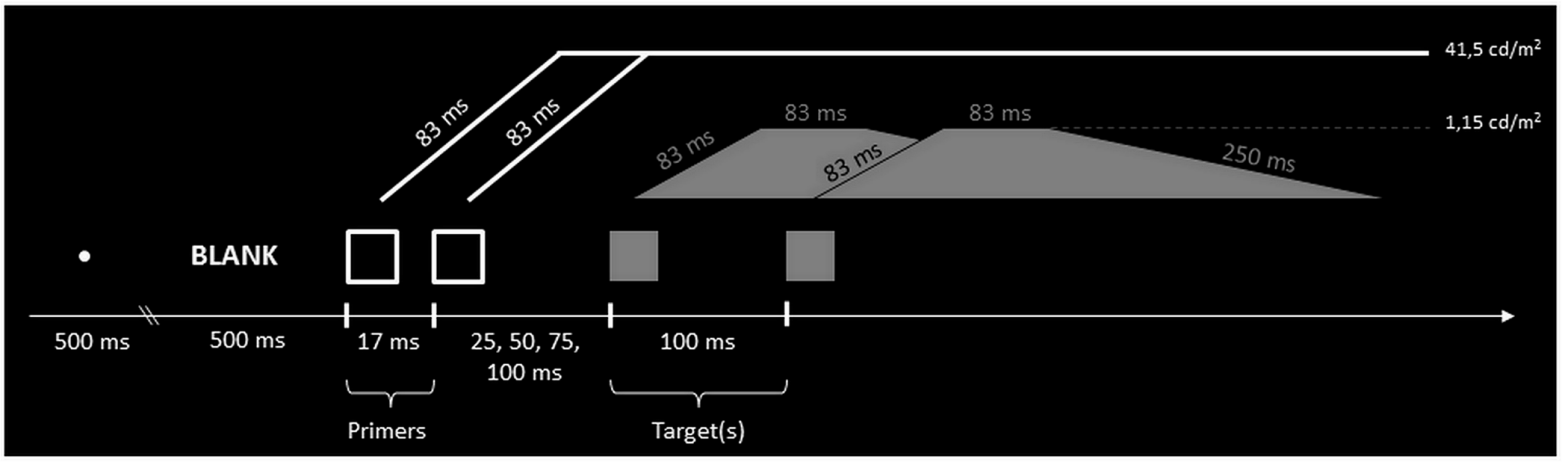
Schematic view of the luminance ramps of primers and targets. In the first task of Experiment 1, the two frames were filled in with a 100 ms onset asynchrony, such that the sequence was unambiguously perceived. Subjects were instructed to press the response button on the same side as the first square. In the second task of Experiment 1 only one frame was filled in, and subjects were told to press the button on the side of the filled-in square.

*Primers*: The primers were two empty square frames of 0.9° * 0.9° of visual angle, with a white contour (contour thickness: 0.06°). They were presented one to the left and one to the right of the central fixation point (a white point of 0.3° of visual angle, 41.5 cd.m^-2^) and separated by 6° of visual angle. The background was black (0.008 cd.m^-2^). The primers appeared either simultaneously or asynchronously. The Stimulus Onset Asynchrony (SOA) of the primers corresponded to two periods of screen refreshing rate, i.e. approximately 17 ms. We expected this asynchrony to be invisible, because it is below the conventional threshold for conscious perception, which is about 30 ms [7,9,21]. The luminance of the frames was gradually increased from 0.02 cd/m² to 41.5 cd/m², within a ramp of 83 ms, with a view to preventing activation of the magnocellular pathway which is sensitive to rapid luminance changes and induces attentional capture [22,23]. When there was a SOA of 17 ms between primers, this asynchrony continued as luminance increased until both primer frames attained their maximum brightness (see Fig 1).

Once the primers appeared on the screen, they stayed there until the end of the trial.

*Targets*: The targets corresponded to the filling-in of the primers. The luminance of the targets was 1.15 cd/m², representing a low level of contrast. We again avoided attentional capture effects by gradually increasing target luminance from 0.02 cd/m² to 1.15 cd/m² with ramp duration of 83 ms. Total target exposure time was 416 ms, including the decreasing luminance ramp of 250 ms. Fig 1 illustrates the luminance ramps of stimuli during the task.

### Procedure for Experiment 1: Temporal order judgment—detection task

**Fig 2:**
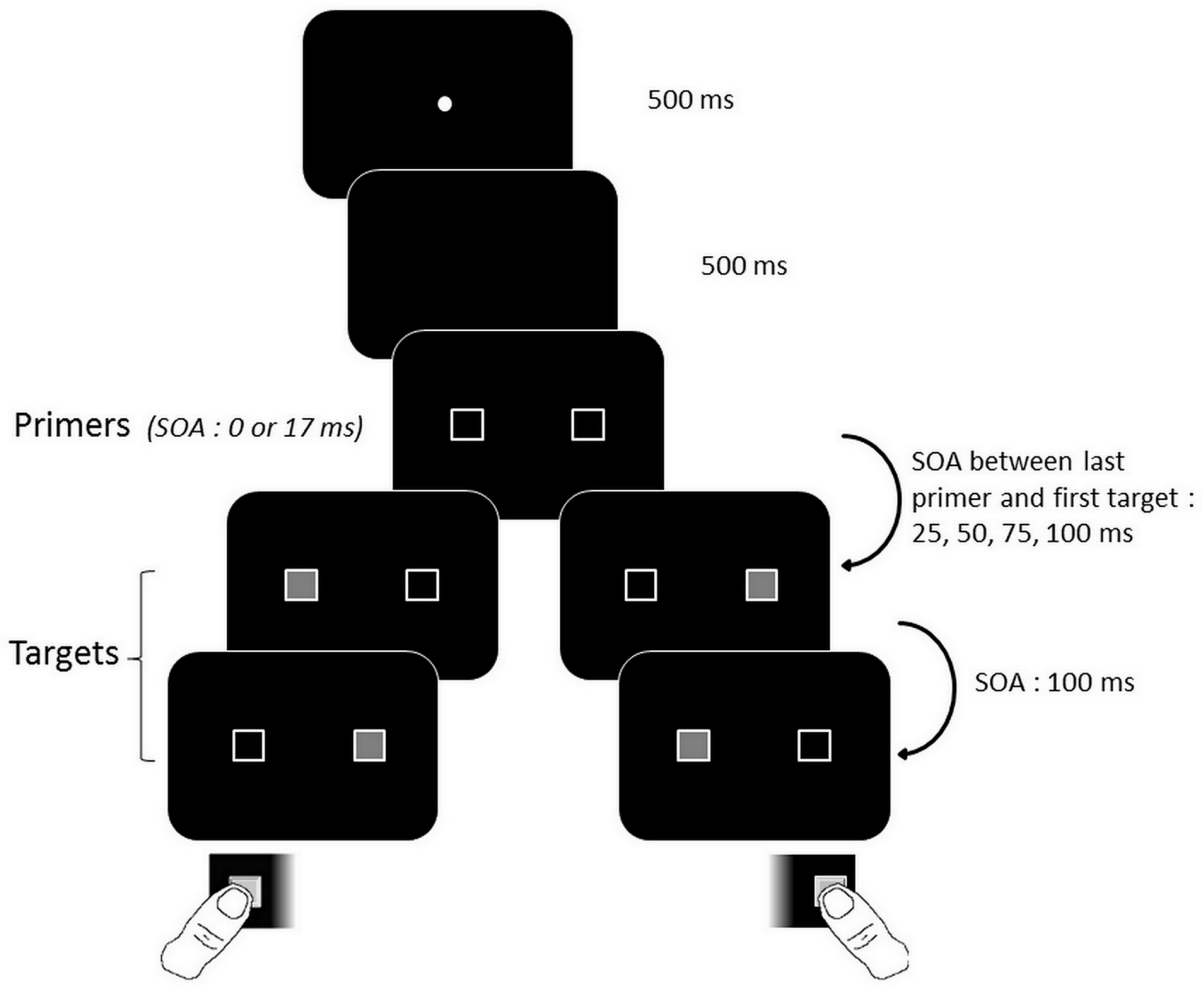
Schematic view of the sequence of events in a single trial in the Temporal Order Judgment in Experiment 1. Primers appeared either simultaneously or asynchronously (left-right or right-left). Targets always appeared asynchronously, in either the left-right or right-left direction. The direction of primers and targets could be either congruent or non-congruent. Subjects responded by pressing the button on the same side as the first target. When the primers were asynchronous, the target stimuli appeared in either the same order as the primers (congruent condition), or the reverse order (non-congruent condition).

In Experiment 1, all subjects started off with a temporal order judgment task (see Fig 2 for protocol details). This task order was chosen to avoid inciting the subjects to rely on the detection of the first target to give their response in the temporal order judgment task.

The test started with the presentation of the central fixation point on the black screen, where it remained for 500 ms. After a further 500 ms delay during which the screen remained black, the primers appeared either simultaneously (Stimulus Onset Asynchrony (SOA) = 0 ms) or asynchronously (SOA = 17 ms). After a further delay of 25 to 100 ms (according to distinct experimental blocks), two target stimuli appeared and filled the inside of the frames. We manipulated the stimulus onset asynchrony (SOA) between the primers and the targets, in order to check for the time course of the primers influence (25, 50, 75 or 100 ms SOA between the last primer and the first target). Each primer-target SOA was used in separate experimental sessions, meaning that this asynchrony was constant within a given session. Targets were always displayed asynchronously, with a SOA of 100 ms. This SOA is long enough to induce a clear perception of sequence. Subjects had to respond as quickly as they could on the same side as the first target (e.g. if the first target stimulus is on the left, they have to press the left button). The response times were then measured.

**Fig 3:**
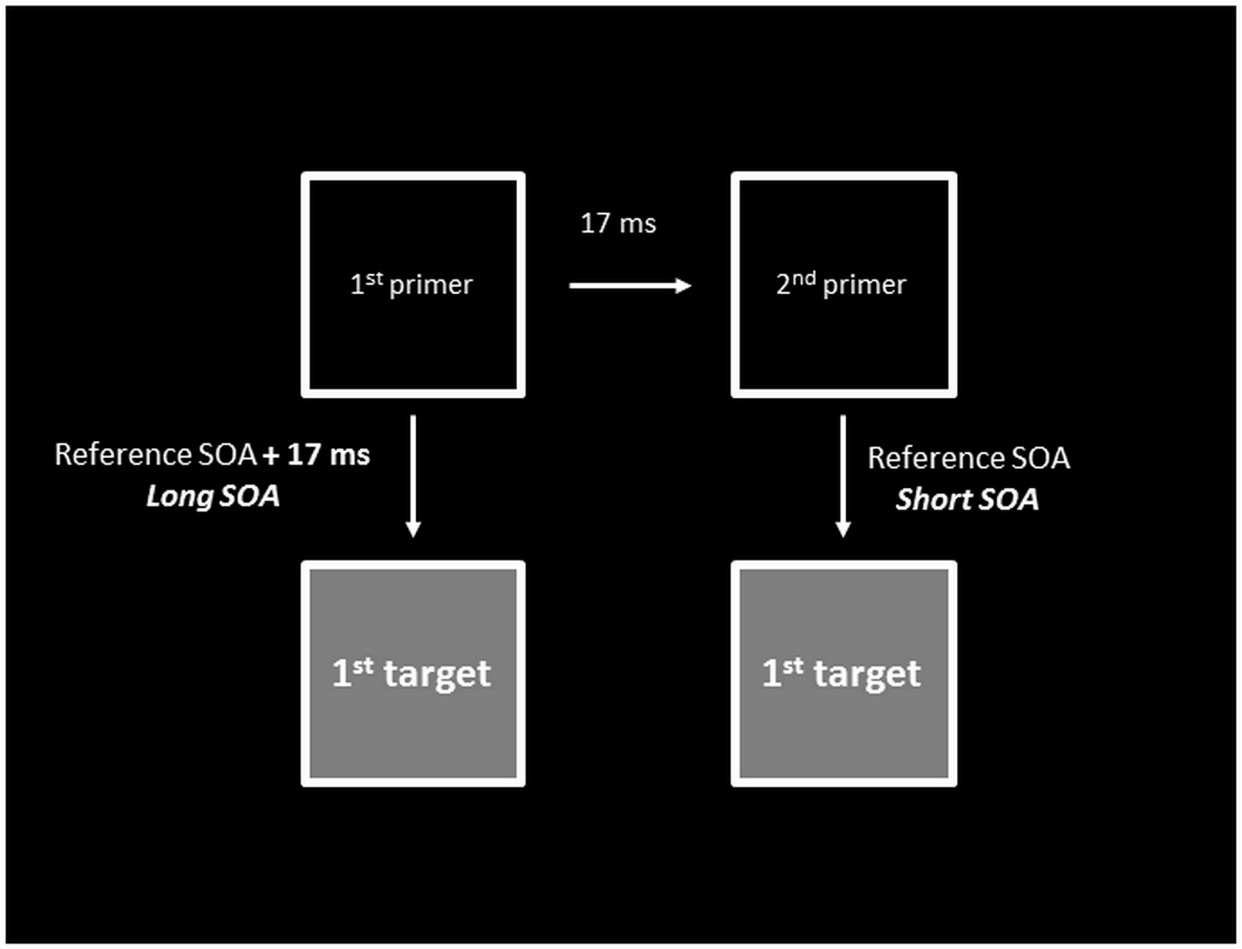
Schematic view of the SOA between the primers and the *first* target. Here we illustrate only the first (or unique) target, to show that in asynchronous conditions, the primer-first target SOA varies as a function of the location of the first target (first or second primer position).

In the asynchronous conditions, the SOA between the primers and targets corresponded to the interval between the onset of the second primer (i.e. the primer which appeared on the screen second) and first target. This meant that when the first target was located on the side of the second primer, it was displayed with a SOA of 25 to 100 ms after this primer (according to the experimental session). In contrast, when the first target was located on the side of the first primer, it was displayed with an additional SOA of 17 ms after the first primer (see details in Fig 3).

To check for this additional time lag, we used two 'simultaneous' sub-conditions in each session, i.e. two sub-conditions with simultaneous primers. One of the sub-conditions had the basic 25 to 100 ms delay between the frames and targets, which thus corresponded to the condition in which the first target was displayed on the side of the second primer. The second ‘simultaneous’ sub-condition had an additional delay of 17 ms (25, 50, 75 or 100 ms plus 17 ms) corresponding to the first target being displayed on the side of the first primer.

There were thus four experimental sessions, each with a different primer-target SOA (25, 50, 75 and 100 ms). In each session, there were four experimental conditions: (1) the condition with asynchronous primers in the same order as targets (congruent condition) was compared to (2) a condition with simultaneous primers, and a primer-target onset asynchrony of 25 to 100 ms with an additional delay of 17 ms (‘long’ control condition); (3) the condition with asynchronous primers in the opposite order to targets (non-congruent condition) was compared to (4) a condition with simultaneous primers, and a primer-target onset asynchrony of 25 to 100 ms (‘short’ control condition).

In each session of Experiment 1, there were 80 trials with simultaneous primers (half with an extra 17 ms primer-target time lag), 40 trials with asynchronous primers in the congruent condition, and 40 trials with asynchronous primers in the non-congruent condition. This led to a total of 160 trials.

In this experiment, all conditions (simultaneous with or without further time lag, asynchronous primers in the congruent or non-congruent condition), and order of primers and targets were equally represented, and displayed in randomized order. The four experimental sessions resulting from the manipulation of the primer-target delay were run in separate blocks, but in random order.

**Fig 4:**
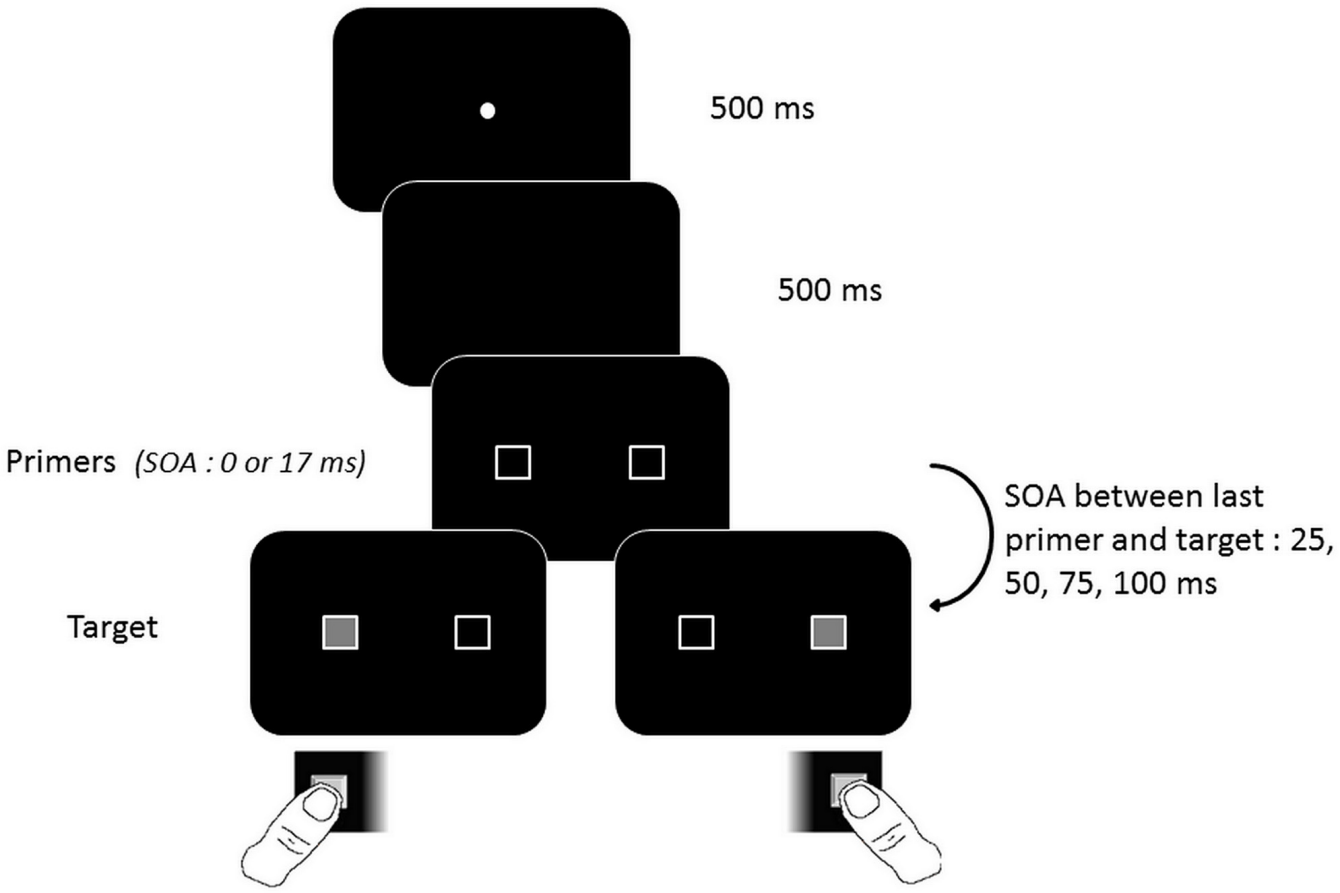
A schematic view of the sequence of events in a single trial in the detection task of Experiment 1. Primers appeared either simultaneously or asynchronously (left-right or right-left direction). A single target then appeared on the side of either the first or second primer. Subjects responded by pressing the button on the side of the target.

After the temporal order task, all subjects ran a detection task (see Fig 4 for protocol details). The main difference was that it used only one target instead of two. Either the right or left frame primer was filled in and represented the target. Subjects had to detect the side of the target and respond to it as quickly as possible. We measured their reaction time.

As for the temporal order judgment task, we manipulated the primer-target SOA, and we had two simultaneous sub-conditions, one with and one without an additional period of 17 ms between the onset of primers and the appearance of the target.

In case of asynchronous primers, the unique target was either on the side of the first or the second primer. There were four main experimental conditions like in the temporal order judgment task, i.e. (1) primers asynchronous and target on the side of the first primer, compared to (2) primers simultaneous and primer-target SOA between 25 and 100 ms with an additional delay of 17 ms, (3) primers asynchronous and target on the side of the second primer, compared to (4) primers simultaneous and primer-target SOA between 25 and 100 ms. The randomization was identical to the first task. However, in order to reduce the duration of the experiment, we used only 24 trials per condition, instead of 40, with a total of 96 instead of 160 trials per session.

#### Statistical analyses

Analyses (repeated measures ANOVAs) were performed using Statistica 8.0 software by StatSoft. The median response time was the dependent variable (given the relatively low number of trials in the detection task).

ANOVAs were used to detect differences between experimental conditions, and post hoc follow-up tests were performed to localize these differences. Our effects were mainly on RTs and rather small, and the number of variables was high, at least in Experiment 1. We thus choose the Bonferroni post-hoc test, which is conservative, in order to reduce type 1 errors (i.e. results falsely interpreted as being significant). There were four within-subject variables, the task (temporal order judgment vs. detection), the primer-target SOA (25, 50, 75 vs 100 ms), the experimental conditions (congruent vs. non congruent in the temporal order judgment task, or target on the side of the 1^st^ vs. 2d primer in the detection task; in case of simultaneous primers this corresponds to long- vs short- simultaneous conditions respectively) and the type of primers (simultaneous vs. asynchronous). A critical analysis was the comparison of the asynchronous conditions with their corresponding simultaneous primers conditions to investigate whether facilitation or impairments effects arise as a result of the primers order, i.e. on the side of the first or second primer. The significance level in the ANOVA was set at α = 0.05, but multiple comparisons were taken care of by using the Bonferroni post-hoc tests.

Error rates were low (<2%), and the analysis of error rates showed there was no trade-off between speed and accuracy. For the sake of simplicity, we only give response times.

### Control task

A control task followed each Experiment. The purpose of it was to test whether participants detected the asynchrony of the primers. For each Experiment, we used the same protocol as described above. In Experiment 1, we used the TOJ protocol for the control task with a primer-target SOA of 100 ms. The primers are expected to be all the more visible that the primer-target SOA is longer. Since the 100 ms was the longest primer-target SOA in our experiments, it is the one in which the probability of seeing the primer asynchrony is the highest. Only the instructions differed from the main tasks. Subjects were informed that the primers were sometimes asynchronous and were asked to respond on the side of the first primer (second primer in Experiment 4) by pressing the corresponding response key, regardless of the appearance of targets. They were instructed to respond randomly if they did not perceive an asynchrony between the primers. This task consisted of 80 trials with simultaneous primers and 80 trials with asynchronous primers, of which 40 had primers in the "left-right" direction (first primer on the left and second primer on the right) and 40 had primers in the opposite direction.

#### Statistical analyses

The control task was a forced-choice task, and we considered only the trials with asynchronous primers. We calculated the correct response rate in the case of asynchrony between primers and ran a t-test to compare it with the theoretical chance rate, i.e. 50%. A significant difference means subjects distinguish between the first and second primer.

## Results of Experiment 1

### Simultaneous condition

**Fig 5:**
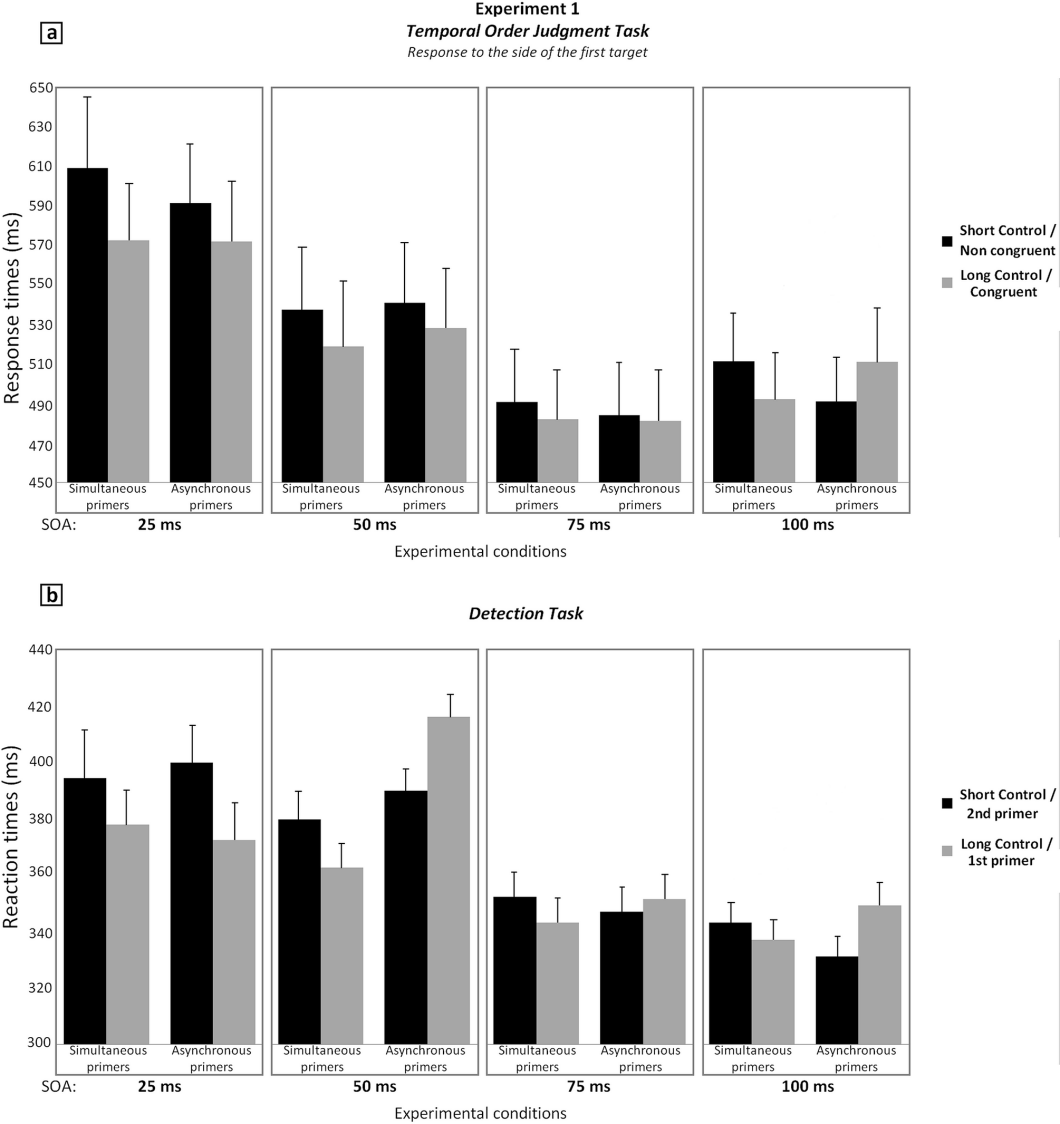
Results of Experiment 1. Response times averaged across subjects in the two experimental tasks: (a) temporal order judgment; (b) detection, as a function of the experimental conditions and of the primer-target SOA. SEMs are shown for each mean. For simultaneous primers, the black histograms correspond to the condition without additional SOA (short-control condition) and the grey histogram to the condition with an additional SOA of 17 ms (long-control condition). For asynchronous primers, the black histogram corresponds to the unique or first target in the location of the second primer (non-congruent condition in case of the TOJ task) and the grey histogram to the unique or first target in the location of the first primer (congruent condition in case of the TOJ task).

We first compared the impact of the additional delay of 17 ms in the simultaneous condition (Fig 5). There were three within-subject variables, the task (temporal order judgment vs. detection), the SOA (25 vs. 50 vs. 75 vs. 100 ms with or without an additional delay of 17 ms) and the delay correction (without vs. with the additional delay of 17 ms). The analysis showed a significant interaction between the SOA and the delay correction (F [3, 33] = 3.28, p<.05, η² = 0.23), without interaction with the task (Fs<1). The Bonferroni post-hoc test shows effects to be significant at the shortest SOAs only. When the SOA was 25 ms, RTs were shorter by 26 ms when there was an additional delay of 17 ms between primers and targets than when there was no additional delay (p<.001). A similar effect of 17 ms was observed for the SOA of 50 ms (p<.01). The effect was smaller and not significant anymore for the delays of 75 ms (p>.9), and 100 ms (12 ms, p>.1). The decrease of this effect with increasing primers-target SOA suggests that the additional 17 ms delay improves preparation effects mainly at short primer-target SOAs.

We took this effect into account, and in the following, systematically compared asynchronous conditions with its corresponding simultaneous condition. As a matter of fact, displaying the first or unique target on the side of the first primer in case of an asynchrony led to an additional primer-target SOA of 17 ms, as compared to the SOA occurring when the target was displayed on the side of the second primer (Fig 2).

### Global analysis

The ANOVA analysis showed several main effects. In particular, RTs were longer for the temporal order judgment than for the detection task, which was to be expected (525 vs. 360 ms, F[1, 11] = 37.3, p<.001, η² = 0.77). Besides, RTs decreased as the primer-target SOA increased (482, 456, 415, and 419 ms for the SOAs of 25, 50, 75, and 100 ms respectively, F[3, 33] = 12.3, p<.001, η² = 0.53). In addition to main effects, there was a significant interaction between the primer-target SOA and the experimental conditions (first or unique target on the side of the 1^st^ vs. 2d primer; in case of simultaneous primers this corresponds to the presence vs. absence of an additional delay of 17 ms respectively, F[3, 33] = 10.1, p<.001, η² = 0.48). There was also an interaction between the primer-target SOA and the type of primers (simultaneous vs. asynchronous, F[3, 33] = 6.1, p<.005, η² = 0.36), and between the experimental conditions and the type of primers (F[1, 11] = 27, p<.001, η² = 0.71). However, we first needed to decipher the 4-level interaction between the task, the SOA, the experimental conditions and the type of primers, F[3, 33] = 4.6, p<.01, η² = 0.3). Despite the high number of variables, the Bonferroni post-hoc test suggested this interaction to result from effects at 25 and 50 ms primer-target SOAs.

### SOA of 25 ms

For the primer-target SOA of 25 ms, the Bonferroni post-hoc analysis showed significant effects in the temporal order judgment task only. However, the main effect was observed for simultaneous primers and resulted from RTs being longer when the primer-target delay was 25 ms only, without further 17 ms delay. This effect has been described in the paragraph ‘simultaneous primers’ and will not be discussed further, especially as this effect at 25 ms unlikely explains the 4-levels interaction, since there was no effect of session in the analysis on simultaneous primers.

### SOA of 50 ms

At the SOA of 50 ms, the Bonferroni post-hoc test showed significant results in the detection session only. RTs observed when the primers are asynchronous and the target on the side of the first primer are slower by 50 ms than in the corresponding control condition with simultaneous primers (with 50+17 ms primer-target SOA, (p<.001), suggesting an impairment on the side of the first primer. There is no significant difference between RTs for asynchronous primers when the target is located on the side of the second primer and RTs in the corresponding simultaneous condition (with a delay of 50 ms, p>.9).

The Bonferroni post-hoc test did not show significant effects for the primer-target SOAs of 75 and 100 ms. However, in the global ANOVA, the four-levels interaction is the only one including the task variable, in contrast with the three two-levels interactions. For example, it is unlikely that the interaction between experimental conditions (first or unique target on the side of the 1^st^ vs. 2d primer, with their corresponding simultaneous condition) and the type of primer (simultaneous vs. asynchronous) is driven solely by the effect in the detection task at 50 ms. The graph suggests that effects also occur at the SOAs of 75/100 ms, which might have been missed due to the large number of variables and type II errors in the Bonferroni post-hoc test We thus performed an additional ANOVA by taking into consideration only the primer-target SOAs of 75 and 100 ms.

### SOAs of 75 and 100 ms

The ANOVA conducted on data observed at 75 and 100 ms primer-target SOAs showed a significant two-levels interaction between experimental conditions (first or unique target on the side of the 1^st^ vs. 2d primer, with their corresponding simultaneous condition) and the type of primer (simultaneous vs. asynchronous), F[1, 11] = 52.7, p<.001, η² = 0.83, and a three-level interaction between these two variables and the primer-target SOA, F[1, 11] = 4.9, p<.05, η² = 0.31. The Bonferroni post-hoc test performed on the three-levels interaction did not show significant results. Hence we focus on the two-levels interaction and average over the two SOAs, albeit results described hereafter are clearer for the 100 ms primer-target SOA. The post-hoc test performed on the two-levels interaction showed that in case of asynchronous primers, RTs were slower when the target was located on the side of the first rather than the second primer (by 10 ms, p<.005). This effect contrasts with the 11 ms RT advantage observed in case of simultaneous primers when the primer-target asynchrony is 75 or 100+17 ms (p<.005). Moreover, RTs observed when the primers are asynchronous and the target on the side of the first primer are slower by 10 ms than in the corresponding control condition with simultaneous primers (with 75 or 100+17 ms primer-target SOA, p<.005), suggesting an impairment on the side of the first primer. On the other hand, RTs for asynchronous primers when the target is located on the side of the second primer, are faster by 11 ms than in the corresponding control condition with simultaneous primers (with a primer-target SOA of 75 or 100 ms, p<.005), suggesting a facilitation effect on the side of the second primer. All data can be found in the datasets (S1 Dataset. Dataset of Experiment 1).

The control experiment was performed immediately after Experiment 1 by using the TOJ protocol with a primer-target SOA of 100 ms. We compared the subjects’ correct response rate of temporal order judgment (TOJ) on asynchronous primers with the theoretical chance rate (50%). The t-test analysis showed no significant difference between the two rates (Mean TOJ on primers = 50.45%; t-value = 0,36; p = 0.72), meaning subjects did not distinguish the first from the second primer.

### Discussion of Experiment 1

The significant differences found in Experiment 1 between the simultaneous and asynchronous conditions suggest that asynchrony affects subject’s judgments. The results attest to the fact that the two asynchronous frames are distinguished in time on an implicit level, i.e. for asynchronies that are not consciously detected. They are consistent with prior evidence based on the Simon effect [4].

The advantage provided by the additional delay of 17 ms in case of simultaneous primers can be explained by an additional preparation time, especially at the shortest delays [24]. When the primer-target SOA is 25 ms, an additional delay of 17 ms represents a very large increase of preparation time. These effects might have obscured the impact of the asynchrony. However, when the effect of this additional delay is reversed for asynchronous primers, like for the SOAs of 50 ms (detection session) and of 75 to 100 ms (detection and temporal order judgment session), it cannot be attributed to this possible confounding effect. A most important effect is the fact that in both sessions and at the SOA of 75 to 100 ms, there is a RT advantage when the unique or first target is displayed on the side of the second primer.

These results suggest that after the display of two stimuli the focus shifts towards the second stimulus [5], which could explain the results we obtained with Experiment 1. If the asynchrony of the primers biases attention towards the second stimulus following its presentation, it could explain why it is that subjects are quicker to respond when the first target appears in the same place as the second primer. The present results are therefore consistent with previous studies, and especially with the idea that subjects are oriented towards the second stimulus after watching a sequence of two. It is also worthy of note that the effects of asynchronous primers do not differ in the detection and temporal order judgment tasks. The detection task does not involve any temporal order judgment, suggesting that the focus on the second primer occurs independently of task instructions. However, the effects were very small. Moreover, in the detection task it might be asked whether this effect is due to the fact that the detection sessions always followed the temporal order judgment sessions. In the following, we ran the detection task alone at the two SOAs that led to the clearest results in Experiment 1, i.e. 50 and 100 ms, and then compared the results with those observed in Experiment 1.

## Experiment 2: Detection of a single target task, SOA 100 ms

### Protocol of Experiment 2

Experiment 2 was strictly identical to the detection task of Experiment 1, when the primer-target SOA was 100 m. This time however, the twelve participants included ran this task exclusively, without temporal order judgment task.

### Statistical analyses

Analyses (repeated measures ANOVAs) were performed in the same way as for Experiment 1, with median reaction time as the dependent variable.

### Results of Experiment 2

**Fig 6:**
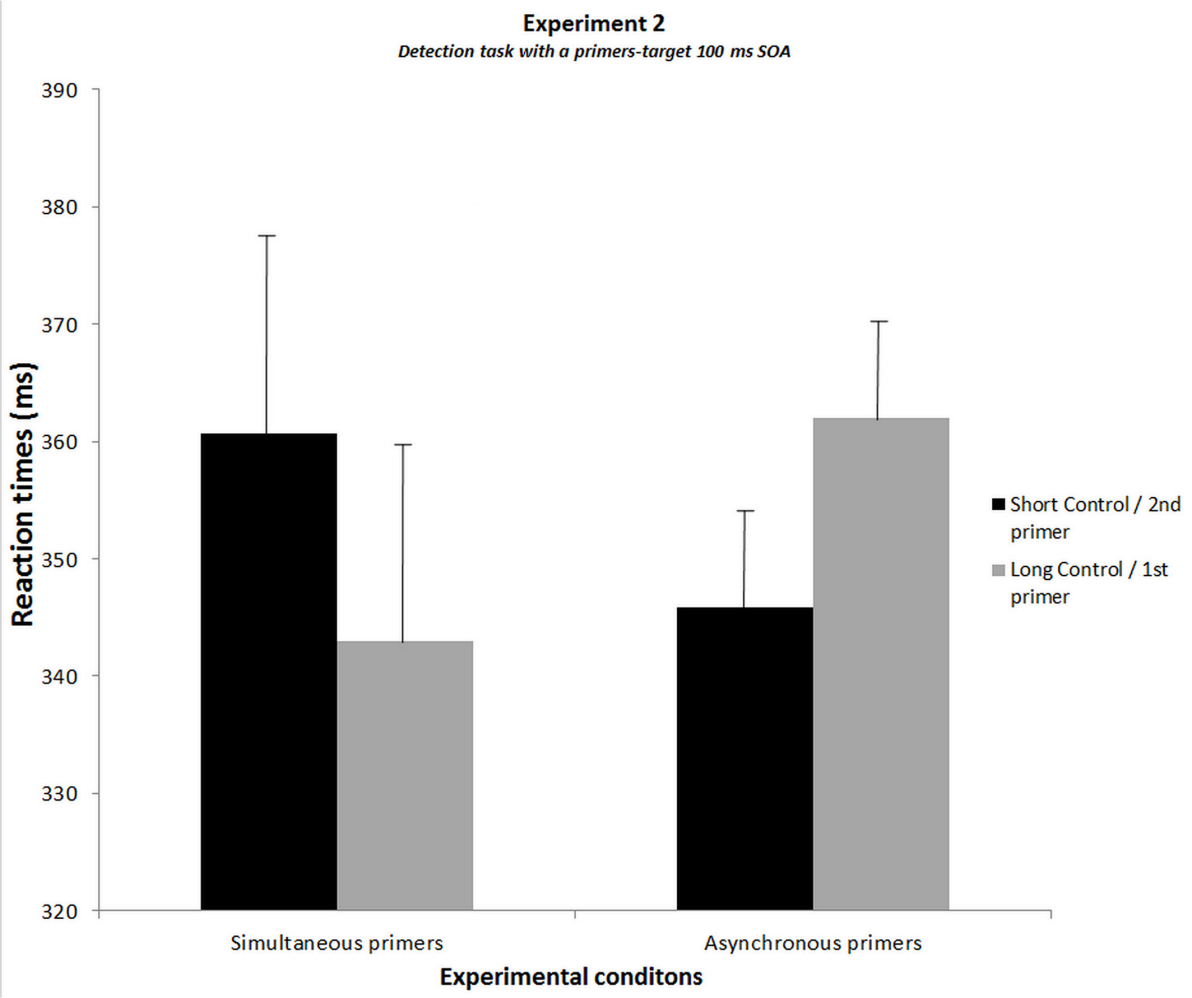
Results of Experiment 2 showing the results of the detection task with 100 ms SOA between primers and target. The graph shows the reaction times averaged over subjects for each experimental condition. For simultaneous primers, the black histogram corresponds to the condition without additional SOA (short-control condition) and the grey histogram to the condition with an additional SOA of 17 ms (long-control condition). For asynchronous primers, the black histogram corresponds to the target in the location of the second primer and the grey histogram to the target in the location of the first primer. SEMs are shown for each mean.

The analysis of variance conducted on the results of the new group of subjects showed a significant interaction between experimental conditions and the type of primers (F[1, 11] = 25.9, p<.001, η² = 0.70) (Fig 6). The Bonferroni post-hoc test showed that in case of asynchronous primers, RTs were slower when the target was located on the side of the first rather than the second primer (by 23 ms, p<.005). In addition, RTs observed when the primers were asynchronous and the target on the side of the first primer were slower by 20 ms than in the corresponding simultaneous condition (with 100+17 ms primer-target SOA, p<.01), suggesting an impairment on the side of the first primer. In contrast RTs for asynchronous primers when the target is located on the side of the second primer did not differ significantly from the corresponding simultaneous condition (p>.1). We then conducted an analysis of variance to compare these results with those observed in Experiment 1 in the detection session with the same SOA (experiment was taken as a between-group factor). There was no significant difference between the two experiments (Fs<1.2, ps>.28). The analysis confirmed a significant interaction between experimental conditions and the type of primers (F[1, 22] = 30.7, p<.001, η² = 0.58). The Bonferroni post-hoc test confirmed all the effects described in Experiment 1. It confirmed in particular the fact that RTs are faster for asynchronous primers when the target is located on the side of the second primer than for the corresponding simultaneous condition with a primer-target SOA of 100 ms (by 12 ms, p<.05), suggesting a facilitation effect on the side of the second primer. All data of Experiment 2 can be found in the datasets (S2 Dataset. Dataset of Experiments 2 & 3).

The control task was performed immediately after Experiment 2 using the same detection task protocol. Subjects were instructed to press on the side of the second primer. We compared the subjects’ correct response rate in case of asynchronous primers with the theoretical chance rate (50%). The t-test analysis showed no significant difference between the two rates (Mean TOJ = 51.41%; t-value = 0.78; p = 0.45), meaning subjects did not distinguish the first from the second primer.

### Discussion of Experiment 2

The results of Experiment 2 confirm that a 17 ms asynchrony influences performance even in a task that requires no temporal judgment, the suggestion being that the asynchrony is processed automatically, independently of the task at hand. The results show that with SOAs of 100 ms, i.e. the same timing parameters as in Experiment 1, subjects are slower to respond when the target appears on the side of the first rather than the second primer. When performance was compared to conditions with simultaneous primers, the impairment on the side of the first primer was the only significant effect. The facilitation on the side of the second primer (relative to its corresponding simultaneous condition) did not reach significance, but did not differ from the one observed in Experiment 1. More generally, there was no significant difference when the task had been preceded (Experiment 1) or not (Experiment 2) by a temporal order judgment task. This suggests these effects to be independent of the instructions. This question was also explored in Experiment 3 for the delay of 50 ms.

## Experiment 3: Detection of a single target task, SOA 50 ms

### Protocol of Experiment 3

Experiment 3 was strictly identical to the detection session of Experiment 1, when the primer-target SOA was 50 ms. Like in Experiment 2, the twelve participants included ran one session only, without temporal order judgment task.

### Results of Experiment 3

**Fig 7:**
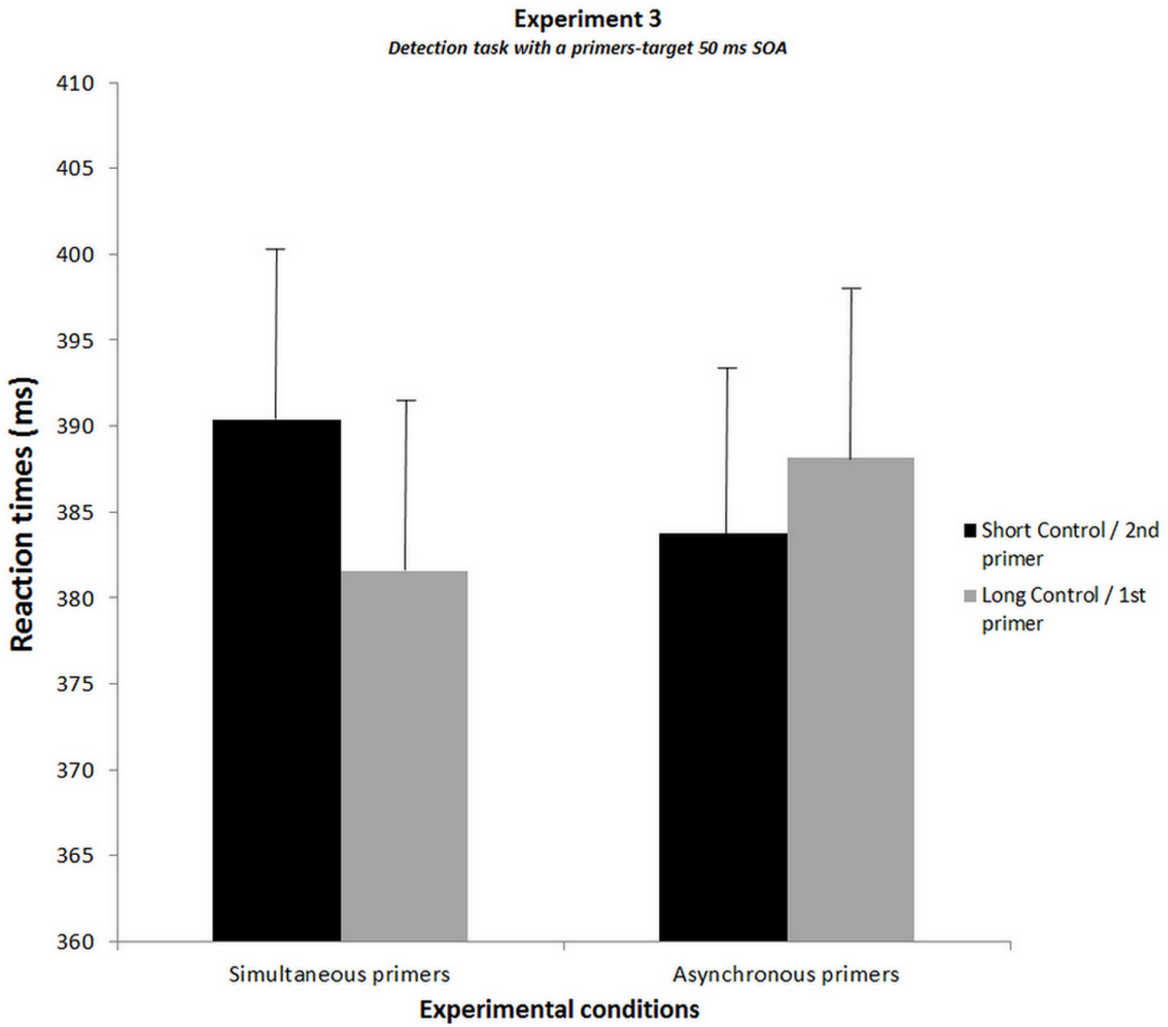
Results of Experiment 3 showing the results of the detection task with 50 ms SOA between primers and target. The graph shows the reaction times averaged over subjects for each experimental condition. For simultaneous primers, the black histogram corresponds to the condition without additional SOA (short-control condition) and the grey histogram to the condition with an additional SOA of 17 ms (long-control condition). For asynchronous primers, the black histogram corresponds to the target in the location of the second primer and the grey histogram to the target in the location of the first primer. SEMs are shown for each mean.

The analysis of variance conducted on the results of the new group of subjects showed a significant interaction between experimental conditions and the type of primers (F[1, 11] = 8.6, p<.05, η² = 0.44) (Fig 7). However, the Bonferroni post-hoc test did not show significant effects.

Moreover, the analysis conducted to compare these results with those observed in Experiment 1 showed significant two-levels interactions (1) between experiments (1 vs. 3) and type of primers (simultaneous vs. asynchronous, F[1, 22] = 17.3, p<.001, η² = 0.44) (2) between experimental conditions and types of primers (F[1, 22] = 21.5, p<.001, η² = 0.49). In addition it showed a significant three-level interaction (3) between experiments (1 vs. 3), experimental conditions and type of primers (F[1, 22] = 5.4, p<.05, η² = 0.2).

The Bonferroni post-hoc test conducted on the three-level interaction mainly confirms the results already described in Experiment 1, and the absence thereof in Experiment 3. All data of Experiment 3 can be found in the datasets (S2 Dataset. Dataset of Experiment 2 & 3).

The control task was performed immediately after Experiment 3 using the same detection task protocol. Subjects were instructed to press on the side of the second primer. We compared the subjects’ correct response rate for asynchronous primers with the theoretical chance rate (50%). The t-test analysis showed no significant difference between the two rates (Mean TOJ = 50.66%; t-value = 0.42; p = 0.68), meaning subjects did not distinguish between the first and second primers.

### Discussion of Experiment 3

The results of Experiment 3 do not confirm the effects observed in Experiment 1 for a primer-target SOA of 50 ms, and on the contrary show a significant difference between the two experiments, suggesting that the impairment on the side of the first primer occurs only in the context of a temporal order judgment task.

All in all, however, the effects we observed in Experiments 1 to 3 were mainly related to either an impairment (i.e. slowed RTs) when the first or unique target was displayed on the side of the first primer, or a facilitation when this target was displayed on the side of the second primer. This means that whenever there is an effect, subjects systematically tend to press on the response key located on the side of the second primer. We wondered whether the hand response played a role in the observed effect, and checked for this possibility by using a temporal order judgment task like in Experiment 1 and asking subjects to press on the side of the second target instead of the first one. This means that Experiments 1 and 4 differ only by the fact that responses are on opposite sides. If the effects observed in preceding experiments are related to hand responses, e.g. a spatial bias generated by the time asynchrony, then they should be reversed in the following experiment. If in contrast, the biases are independent of such visuomotor mechanisms, then effects should be similar as in Experiment 1.

## Experiment 4: Temporal order judgment task, SOA 100 ms.

### Protocol of Experiment 4

Experiment 4 was strictly identical to the temporal order judgment task of Experiment 1, when the primer-target SOA was 100 ms, with the exception of the instruction. This time, subjects were instructed to press on the side of the second target. Two different groups of twelve participants were included, who ran one session only.

### Results of Experiment 4

**Fig 8:**
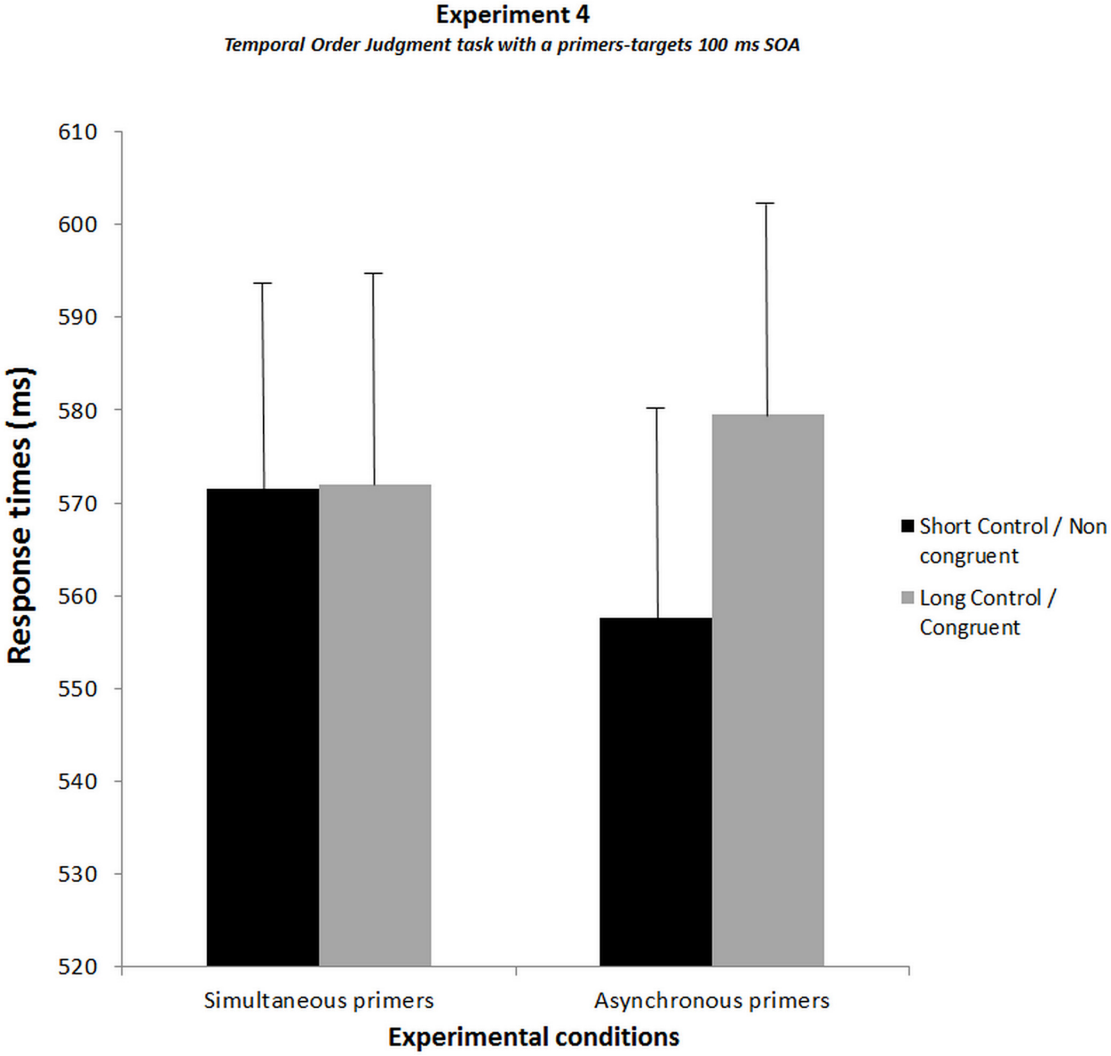
Results of Experiment 4 showing the results of the temporal order judgment task with 100 ms SOA between primers and target. The graph shows the response times averaged over the two groups of subjects for each experimental condition. For simultaneous primers, the black histogram corresponds to the condition without additional SOA (short-control condition) and the grey histogram to the condition with an additional SOA of 17 ms (long-control condition). For asynchronous primers, the black histogram corresponds to the first target in the location of the second primer (non-congruent condition) and the grey histogram to the first target in the location of the first primer (congruent condition). SEMs are shown for each mean.

The results did not interact with groups (Fs<1.7, ps>.2) and will be displayed averaged over the two groups (Fig 8). There was a significant interaction between experimental conditions and the type of primers (F[1, 23] = 8.7, p<.01, η² = 0.27). In this experiment the two simultaneous conditions led to identical results (572 ms), but the Bonferroni post-hoc test showed that in case of asynchronous primers, RTs were significantly slower when the first target was located on the side of the first primer (congruent condition) rather than the second primer (non-congruent condition) (by 22 ms, p<.005). In addition, RTs tended to be faster for asynchronous primers when the first target was located on the side of the second primer (non congruent condition) than in the corresponding simultaneous condition (by 14 ms, p =. 08).

When compared with the results of Experiment 1 with a SOA of 100 ms, there was no significant interaction with experiments (Fs<1) and results confirmed a significant interaction between experimental conditions and the type of primers (F [1, 34] = 23.2, p<.001, η² = 0.41). The Bonferroni post-hoc test conducted on this interaction confirmed that in case of asynchronous primers, RTs were significantly slower when the first target was located on the side of the first primer (congruent condition) rather than the second primer (non-congruent condition) (by 21 ms, p<.001). In addition, RTs were significantly faster for asynchronous primers when the first target was located on the side of the second primer (non congruent condition) than in the corresponding simultaneous condition (by 16 ms, p<.005), thus suggesting a facilitation effect on the side of the second primer. All data of Experiment 4 can be found in the datasets (S3 Dataset. Dataset of Experiment 4).

The control task was performed immediately after Experiment 2. Subjects were instructed to press on the side of the second primer. We compared the subjects’ correct response rate for asynchronous primers with the theoretical chance rate (50%). The t-test analysis showed no significant difference between the two rates (Mean TOJ = 51.83%; t-value = 1.68; p = 0.10), meaning subjects did not distinguish between the first and second primers.

### Discussion of Experiment 4

The results of Experiment 4 replicate those observed in Experiment 1. Like in preceding experiments there was an RT advantage when the first target appeared on the side of the second rather than the first primer. This occurred even as the subjects had to press on opposite sides relative to Experiment 1. The effect appears thus to be independent of the hand response. In addition this occurs even as there is no effect of the primer-target SOA manipulation in the simultaneous conditions, suggesting that the main effect is independent of any preparation effect.

## Discussion

**O**ur aim with the present study was to explore how events are coded within elementary time windows. We replicated in our control experiments typical results showing that subjects do not consciously distinguish a first from a second stimulus for 17 ms asynchronies [1,2,7,10,12,25]. Nonetheless, what our results show first and foremost is that the asynchrony is processed automatically and influences subjects’ responses. Both Experiments 1, 2 and 4 show that a 17 ms asynchrony between primers influences performance, confirming thus that stimuli are not processed as if they were co-temporal when there is a time-lag of less than 20–30 ms between them. The results also allow us to further assess how events’ time structure is coded within functional windows.

First of all, Experiments 1, 2 and 4 consistently show an RT advantage when the first or unique target is in the location of the second rather than the first primer, when the primer-target delay is 100 ms. These results replicate those obtained in previous studies with the Simon effect [4,5], which also showed a bias to the side of the second stimulus, even when it was separated from the first one by a sub-threshold SOA. The present paradigm provides some additional clues about how information is processed across very short time scales.

First, the bias to the side of the second primer was systematically observed at a primer-target of 100 ms, even when the target was unique (Experiment 1), and when the task did not involve any temporal order judgment (Experiment 2) showing this effect to be independent of the task context. Task context had an effect only in the detection task, and only when the primer-target SOA was 50 ms (Experiment 3). This suggests contextual influences cannot be excluded and should be further investigated. However, they did not reach significance for SOAs of 100 ms.

The results observed with temporal order judgment tasks further suggest that the effects are not due to automatic priming of direction. As shown by control experiments, primers did not elicit any perception of direction or motion. However, we still had to check whether they activated a subliminal sense of direction, which might have primed the perception of temporal order between targets [18,19]. The fact that performance is hampered rather than facilitated when the primers and targets share the same order confirms the primers do not activate a sense of direction (left-right or right-left), or at least this has no effect in the present experiments.

Another important point is the similarity of the results in the detection task and also in the two temporal order judgment tasks, when subjects press on the side of the first or second stimulus. The temporal asynchrony between primers may indeed have induced a spatial bias similar to the one observed for the Simon effect [13]. For example, the earlier occurring primer was also displayed 17 ms longer in its location than the second primer. This might have primed the spatial location of the first primer. There was certainly a global advantage for simultaneous conditions when there was an additional delay of 17 ms between primers and target, showing that such short delays can have an effect. However, in case of asynchronous primers, such effects should have resulted in a bias on the side of the first primer. This is what was observed in Experiment 4, when subjects had to make a temporal order judgment and to press on the side of the second target. RTs were faster in the non congruent condition, i.e. when the subjects pressed on the side of the first primer. In contrast however, when they had to press on the side of the first target, RTs were still shorter in the non congruent condition, but this time, these responses corresponded to a key press on the side of the second primer. Similarly, in the detection task, response times were shorter when the response was on the side of the second primer. These results show that the bias effects are not related to a physical property of either the first of second primer, and are not a direct response to one of the primer. It is rather an indirect consequence of the primers asynchrony.

To go further, we tried to distinguish between facilitation and impairment effects, although effects were rather small. In Experiments 1 and 4, RTs were (slightly) shorter when the first or unique target was displayed on the side of the second primer, as compared to the corresponding simultaneous condition. This cannot be explained by the effect observed with simultaneous primers since, in Experiment 4, there was no effect of the delay between simultaneous primers and targets. There were also some impairment effects in Experiments 1 and 2 when the target was displayed on the side of the first primer. This suggests that by 100 ms after the display of the primers, there is both an impairment of the information processing in the location of the first primer, and a facilitation in the location of the second primer. We did not observe any significant facilitation in the location of the first primer at earlier delays, but this might have been obscured by confounding effects of the preparation time.

Furthermore, several results suggest these effects may be attentional in nature. What Experiments 1 and 4 show is that subjects are quicker to judge the order of targets when the first target is in the position of the second rather than the first primer. The results are consistent with a prior entry effect induced by the second primer. The prior entry typically entails the presentation of a cue attracting the attention of the subjects in its spatial location. Two stimuli are then displayed successively, and the attention cue facilitates temporal order judgment when its location corresponds to the first stimulus (review in [20]). Even though the mechanisms of the prior entry are discussed, with an effect at either the sensory or decisional level [26], what is important here is that it is mediated by an attention cue. It thus suggests that the ‘weight’ attached to the second primer is sufficient to act as an attentional cue directed towards the first target in Experiments 1 and 4. The fact that the facilitation effect is observed only when there is a primer-target delay of 100 ms suggests attention is displaced towards the second primer by this delay. This is consistent with studies showing that attention shifts within the time range of 50 to 150 ms [27]. Moreover, the fact that the facilitation effect is independent of the hand movement is also an argument in favor of an attention effect rather than an automatic visuomotor effect. It is to be noted that it does not exclude a role of eye movements. Inasmuch eye movements are closely linked with attention shifts [27–29] an association with saccades would still be consistent with the hypothesis of an attention shift.

Such an interpretation remains to be confirmed, given the small amplitude of the facilitation effects. Besides, there is a major difference in relation to the typical literature pertaining to attention, insofar as, in our case, the attention displacement relies on non-conscious information. The present results may involve mechanisms similar to those responsible for typical attention displacement but be triggered by an unconscious cue. It may thus be akin to phenomena described in blindsight, i.e. attention without awareness [30]. Whatever the nature of the mechanisms involved, what is important here is that subjects are able to distinguish between, and follow events over shorter timescales than previously thought. This could have implications for understanding the temporal window and sense of time continuity.

### Implications for the temporal window concept and the sense of time continuity

As mentioned above, the temporal window is defined as a time slot within which all events are processed as if they were co-temporal. Our results show that this definition holds true for subjective judgments but not for implicit processing. Both Experiments 1 and 2 show that events are distinguished in time on an implicit level, independently of the task at hand. Nor is it an artefactual result related to automatic hand responses. On the contrary, it is as if subjects are able to move their attentional focus towards the second stimulus in a sequence of two, even when they are unaware of any asynchrony between these events. This move towards the second stimulus is not straightforward. The 17 ms delay between the primers means processing of the first stimulus has not been completed by the time the second stimulus occurs. The latency of the signal in V1 is about 40 ms [31–33]. Despite this, the second stimulus is not only encoded and distinguished in time from the first one, but the focus can also be moved towards this second stimulus. Even if the move towards the second stimulus involves attention, the attentional shift itself is not a conscious one. Otherwise it would have resulted in conscious perception of the asynchrony of the primers, which is not the case. In fact, this suggests that the brain system is permanently prepared to process new information, as if expecting future events. There are a number of mechanisms described in models such as the predictive coding [34,35] or forward models [36] which could subtend this phenomenon. For example, it could stem from the existence of predictive loops which allow us continuously to check for and anticipate incoming information. The mere existence of such loops and their importance in information processing, i.e. the fact that we permanently look for new events, might be enough to induce a prioritization of fresh incoming information. Such automatic prioritization might in turn induce a mobilization of processes subtending attention and a shift in the attentional focus. In all, implicit mechanisms would allow for a processing of information that takes into account asynchronies and discontinuities at a higher temporal resolution than at the subjective level.

## Conclusions

All in all, the results suggest that on an automatic level, information is distinguished in time over shorter intervals than suggested by experiments based on subjective judgments. This would automatically trigger attentional shifts. On an implicit level, information would thus flow in an almost uninterrupted fashion. This ability to distinguish between events in time may reveal a basic mechanism underlying our ability to go with the flow of time, and may represent the first step towards the coding of events’ time structure and the integration of events over time. The latter would then occur on a subjective level. At this subjective level, we would ignore the asynchrony of events that are close in time, enabling the perception of ‘moments’ in which information is integrated and subjectively perceived to be simultaneous. In other words, our sense of continuity might stem from the implicit processing of successive events, whereas our sense or present would emerge at the subjective level.

It may be this complex hierarchy of mechanisms which underlies our ability to have a sense of both time continuity and subjective present.

## Supporting Information

S1 Dataset Dataset for Experiment 1. This table shows median response times and error rates per experimental condition for each subject in the temporal order judgment task (TOJ) and the detection task. In this task, the SOA between the last primer and the first (or single) target is 25, 50, 75 or 100 ms in separate sessions. The subjects (see Table 1 for details) ran 40 trials per condition in each session of the TOJ task and 24 trials per condition in each session of the detection task. The control task was run with the TOJ protocol (primers-target SOA of 100 ms). Subjects were instructed to respond to the side of the first primer. The rate of correct answers is calculated on conditions with asynchronous primers.
(XLS)

S2 Dataset Dataset for Experiments 2 & 3. This table shows median reaction times and error rates per experimental condition for each subject in the detection task. In this task, the SOA between the last primer and the target is 100 ms in one session and 50 ms in another session. Two groups of subjects (see Table 1 for details) ran 40 trials per condition in one single session. Each experiment was followed by a control task with the same protocol. Subjects were instructed to respond to the side of the second primer. The rate of correct answers is calculated on conditions with asynchronous primers.
(XLS)

S3 Dataset Dataset for Experiment 4. This table shows median response times and error rates per experimental condition for each subject in the temporal order judgment task. In this task, the SOA between the last primer and the first target is 100 ms. Two groups of subjects (see Table 1 for details) ran 40 trials per condition in one session. The control task was run with the same TOJ protocol. Subjects were instructed to respond to the side of the second primer. The rate of correct answers is calculated on conditions with asynchronous primers.
(XLS)
